# The role of chemokines and their receptors in cardiovascular diseases

**DOI:** 10.3389/fimmu.2026.1758588

**Published:** 2026-02-04

**Authors:** Sashwath Srikanth, Louis DeVito, Nicholas J. Constantinesco, Radha Gopal

**Affiliations:** Department of Pediatrics, UPMC Children’s Hospital of Pittsburgh, Pittsburgh, PA, United States

**Keywords:** arrhythmia, atherosclerosis, chemokines, coronary artery disease, heart failure, myocarditis

## Abstract

Cardiovascular diseases (CVD) are increasingly recognized as an outcome of multiple inflammatory processes. Despite a large recent research focus, the majority of attempts to therapeutically target chemokines, have not been successful. Thus, a better understanding of the mechanisms involving chemokines contributing to CVD is warranted to identify the missing gaps in the field. This review summarizes the latest developments on the role of chemokines in the pathogenesis of atherosclerosis, heart failure (HF), coronary artery disease, myocarditis, cardiomyopathy, and arrhythmias. We also aim to provide insight into the current clinical trials and potential therapeutics targeting chemokines and their receptors.

## Introduction

Chemokines are molecules that play a pivotal role in the process of inflammation. Chemokines are essentially low molecular weight chemotactic cytokines (8–10 kd) with 20–70 percent homology (1). Based on the relative position of their cysteine residues, they are classified into four families (C, CC, CXC, CX3C) (2, 3). Out of the four families, the two families (α and β) that have four cysteine residues have been extensively studied. In the α family the first two cysteine residues are separated by an amino acid (CXC) whereas in the β family, the two cysteine residues are adjacent to each other (CC). Fractalkine is another family where there are three amino acids in between the two cysteine residues (CX3C) (4). The fourth family consists of lymphotactin which contains only one disulfide bond (5, 6). The α family can further be divided into those that have and do not have the glutamic acid-leucine-arginine sequence near the N-terminal, prior to the CXC sequence (7). Those that have this sequence play a role in neutrophil chemotaxis (1). Those α-chemokines that do not have this sequence play a role in lymphocyte chemotaxis (8, 9). The β-chemokines can be further sub-divided into two groups: monocyte-chemoattractant protein and eotaxin groups (10). Although most chemokines in the β family contain four cysteines, some of them contain six cysteine residues (C6-CC) (11, 12).

Chemokine receptors are G protein-coupled receptors that have seven transmembrane domains. These receptors interact with specific chemokine ligands and induce a calcium influx. This causes a cellular response that results in chemotaxis of leucocytes. So far 20 different chemokine receptors have been identified in humans. The activation of chemokine receptors on leucocytes has been shown to lead to proliferation, survival, differentiation, cytokine production, degranulation, and respiratory burst (13, 14) (**Table 1**). Recent work has broadened our understanding of the functional versatility and robustness of the chemokine network and its role in the pathogenesis of various diseases.

**Table 1:** Chemokines and their receptor functions.

No.	Chemokines	Chemokine receptors	Primary functions
1	CCL3 (MIP-1α), CCL5 (RANTES), CCL7 (MCP3, CCL13 (MCP4), CCL14 (HCC1), CCL15 (LKN1) and CCL23 (MPIF-1)	CCR1	Monocyte and T cell recruitment and inflammation (112–114).
2	CCL2 (MCP1), CCL7 (MCP3), CCL13 (MCP14)	CCR2	Monocyte migration (113, 114)
3	CCL11 (Eotaxin), CCL24, CCL26	CCR3	Th2 immune response, Eosinophil recruitment, allergy/asthma (114, 115)
4	CCL17 (TARC), CCL22	CCR4	Th2 cell trafficking, skin homing (114–116)
5	CCL3, CCL4 (MIP-1β), CCL5	CCR5	T cell and macrophage migration, Co-receptor for HIV infection (114, 117, 118)
6	CCL20	CCR6	Th17 cell and dendritic cell recruitment, psoriasis (114, 119)
7	CCL19, CCL21	CCR7	T cell and dendritic cell migration to the lymph nodes and intestinal Peyer’s patches (114, 120)
8	CCL1	CCR8	Recruitment of Tregs (114, 121)
9	CCL25	CCR9	T cell homing to gut mucosa (122) (114)
10	CCL27, CCL28	CCR10	Skin and mucosal immune response (114, 123)
11	CXCL8 (IL-8)	CXCR1	Neutrophil recruitment (114, 124)
12	CXCL1, CXCL2, CXCL8	CXCR2	Neutrophil recruitment, angiogenesis (114, 124, 125)
13	CXCL9 (MIG), CXCL10 (IP-10), CXCL11	CXCR3	Th1 cell trafficking (114, 126, 127)
14	CXCL12 (SDF-1)	CXCR4	T cell and stem cell homing, HIV co-receptor (114, 128)
15	CXCL13	CXCR5	Trafficking and homing of B cells (114, 129, 130)
16	CXCL16	CXCR6	T cell migration (131, 132)
17	CXCL12, CXCL11	CXCR7 (ACKR3)	Atypical receptor, scavenger receptor (114, 133, 134)
18	XCL1, XCL2	XCR1	Dendritic cell antigen cross presentation (114, 135)
19	CX3CL1 (Fractalkine)	CX3CR1	Monocyte recruitment (38, 39, 47, 114)
20	Variety of CC and CXC chemokines	ACKR1 (DARC)	Atypical receptor, Angiogenesis and neuroinflammation (114, 136–138)

Cardiovascular disease (CVD) is one of the most common causes of mortality in developed countries. About 655,000 Americans die from heart disease each year – that’s one in every 4 deaths (15). Chemokines play a role in CVDs such as atherosclerosis, coronary artery disease, heart failure, arrhythmias, and myocarditis. The role of chemokines and their receptors in these diseases will be reviewed herein, with a particular focus on the pathogenic mechanism involved in these diseases. We further explored the therapeutic potential of targeting chemokines on CVD.

## Chemokines and their receptors in atherosclerosis

### Atherosclerosis initiation

Atherosclerosis is one of the major causes of CVD including myocardial infarction, stroke, and heart failure. It is a well-known fact that the process of atherosclerosis starts with chronic endothelial damage. This leads to endothelial dysfunction where there is an increased permeability of the endothelial layer. Lipids, especially low density lipoprotein, infiltrate the endothelium and accumulate in the intimal layer of the artery (16). The trapped LDL in the sub-endothelial (intimal) layer of the artery is prone to oxidization and forms oxidized LDL (ox-LDL) (17). Ox-LDL activates macrophages and endothelial cells and gets taken up by macrophages where they get hydrolyzed into free fatty acids and cholesterol (18). However, the free cholesterol undergoes re-esterification and forms cholesteryl esters (19). Macrophages, unable to excrete these esters transform into foam cells, which is one of the first changes noticed in atherosclerosis. Studies have shown that CC and CXC type of chemokines play a role in atherosclerosis initiation.

**Figure 1:**
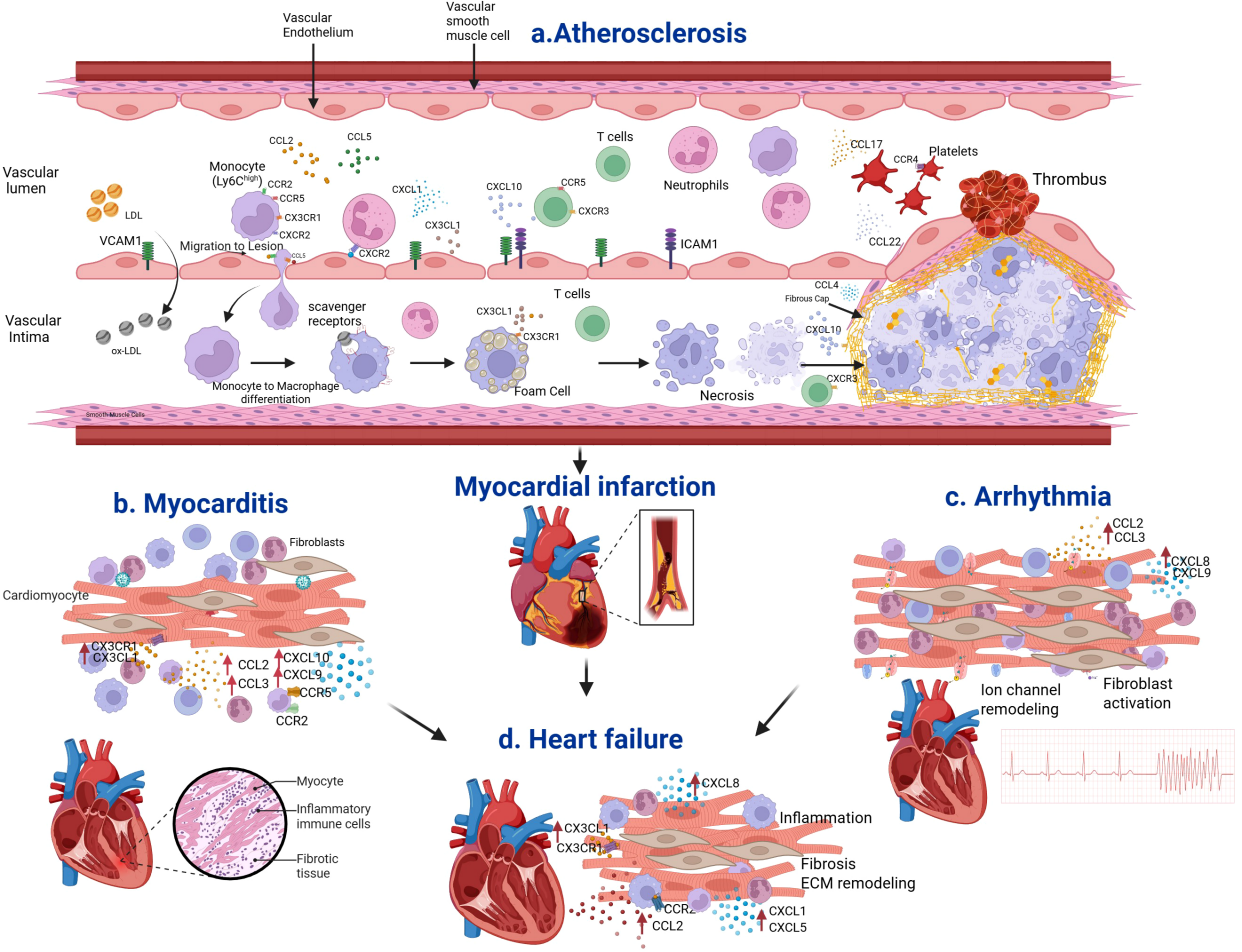
Chemokines and their receptors in atherosclerosis. **(a)** Atherosclerosis disease process is initiated by endothelial dysfunction and accumulation of low-density lipoprotein (LDL) in the sub-endothelial space. LDL is oxidized, and Ox-LDL stimulates the vascular endothelium to increase adhesion molecules, and chemokines such as CCL2, CCL5, CXCL1, and CX3CL1. These chemokines act through the respective receptors and recruit monocytes, neutrophils, and lymphocytes in the sub-endothelium. Macrophages increase the expression of scavenger receptors, engulf ox-LDL, and become foam cells. CX3CL1 acts through CX3CR1 and increases foam cell formation. Over time, foam cells undergo apoptosis and necrosis, thus leading to the accumulation of cell debris and the formation of a necrotic core within the intima. Smooth muscle cells then synthesize collagen and elastin, which form the fibrous cap that covers the necrotic core. If the fibrous cap is fragile, it may rupture and cause coronary artery disease, including MI, stroke, and heart failure. CCL4 and CXCL10 increase plaque instability that increases thrombosis. CCL17 and CCL22 induce platelet activation through their CCR4 receptor. **(b)** Chemokines CCL2, CCL3, CXCL9, CXCL10 and CX3CR1 recruit monocytes, macrophages and neutrophils in myocarditis. **(c)** Chemokines CCL2, CCL3, CXCL8, and CXCL9 drives cellular infiltration, iron channel dysfunction and fibroblast activation in arrhythmia. **(d4)** Chemokines CCL2, CXCL1, CXCL5, CX3CR1 recruit inflammatory monocytes and neutrophils into the injured ventricle, induce fibrosis and structural remodeling in heart failure.

Endothelial cells, upon activation by ox-LDL, release a variety of chemokines that recruit neutrophils and monocytes to the sub-endothelium and the formation of foam cells that initiate plaque growth (20–25) (**Figure 1**). Schober et al. showed that there is an up-regulation of CCL2 in serum and the vessel wall when there is injury to the carotid artery in apolipoprotein E deficient (ApoE^-/-^) mice (26). Monocytes of the classical type (Ly6C^high^) migrate towards the atherosclerotic lesion. One of the key chemokines that attract these monocytes is CCL2. Recently, the CCL2-CCR2 axis has been examined as a drug target for atherosclerosis because of this chemotactic property (27). CCL3 acts through CCR1 and CCR5 and increases the recruitment of neutrophils, thus increasing atherosclerosis (28). CCL4-CCR5 interactions increase endothelial and macrophage cell activation, thereby increasing plaque formation (29). CCR1/5 cause recruitment of monocytes and other circulating cells (30). CCL5 allows for leukocyte attraction to the damaged vascular endothelium, which again causes inflammation (31). CXCL1 and CXCL2 recruit neutrophils and monocytes to the site of inflammation via the receptor CXCR2 (32–36). Studies have shown that a reduction in CXCL1 levels can reduce inflammation and plaque formation (33). CXCL8 is involved in recruitment of neutrophils and monocytes to the endothelium through CXCR2. Studies have shown that 27-oxygenated cholesterol induces CXCL8 in macrophages (37). CX3CL1 has been shown to be involved in macrophage infiltration and lesion formation (38). Classical Ly6C^hi^ monocytes depend on CCR2 or CX3CR1 to enter into atherosclerotic plaques (39). Another study has shown that CCR1 and CCR5 receptors play a role in mediating monocyte infiltration in atherosclerosis (33). Overall, these studies show the chemokines and the receptors are involved in atherosclerosis initiation by recruiting leukocytes to the sub-endothelium thereby enhancing lesion progression (**Figure 1**).

Bacterial and viral infection increases atherosclerosis by directly infecting vascular cells or indirectly increasing inflammatory cytokines and chemokines systemically. Studies have shown that the *Chlamydia pneumoniae* increases CCL2 systemically in HFD-induced atherosclerosis (40). Also, We and others have shown that the influenza infection increases CCL2, CCL3 and CCL5 in the aorta and the serum in Apoe^-/-^ mice (41, 42). These studies suggest that chemokine induction during the infection may be involved in infection-induced exacerbation of atherosclerosis. However, the specific role of these chemokines in infection-induced exacerbation of atherosclerosis is not clear.

### Atherosclerosis progression and regression

As more lipids and debris continue to accumulate, the imbalance between the apoptosis and efferocytosis of macrophages leads to formation of a necrotic core (43–46). Chemokines have been shown to play a role in these processes. Increased apoptosis of monocytes and foam cells were observed in CX_3_CR1-deficient mice suggesting that CX_3_CR1 has a role in cell survival, thereby increasing plaque formation (47). In advanced plaques, fibroblast−activating chemokines such as CCL2, CCL5, CXCL10, and CXCL12 contributing to collagen deposition and fibrous tissue formation (48–50). Rupture of the fibrous cap leads to accelerated thrombosis leading to occlusion of the blood vessel and results in a cardiovascular event. Thus, the thickness of the fibrous cap is crucial for plaque stability. It has been shown that inhibition of CXCL10 leads to stable atherosclerotic plaques suggesting that CXCL10 is involved in plaque destabilization (50). Further, a study has shown that the CXCL10 increases atherosclerosis by modulating balance between Treg and effector T cells, locally (51). Another study has shown that CCL4 antibody treatment increases fibrous cap thickness suggesting that CCL4 is involved in unstable plaque formation (29). Recently it has been shown that CCL2 is a biomarker for plaque instability in patients who underwent carotid endarterectomy (52). As discussed above, a plaque rupture causes an accelerated thrombus formation, and platelets play a crucial role in this process. CCL17 and CCL22 have been shown to induce platelet activation through the CCR4 receptor (53, 54). CXCL12 induces platelet activation and aggregation through CXCR4 expressed on platelets (48). Moreover, migration and transmigration of platelets have been observed to be stimulated by CXCL12 (55). Chemokines are also involved in atherosclerotic plaque regression by immune cell egress, resulting in resolution and tissue remodeling. Trogan et al. showed that CCR7 has a functional significance in atherosclerosis regression. The authors also noticed that regression of the atherosclerotic lesion is inhibited by treatment with antibodies to CCR7 ligands CCL19 and CCL21 (56). Another study has shown that inhibition of CXCL1-CXCR2 and CX3CL1-CX3CR1 axis affected endothelial progenitor cell-mediated atherosclerosis regression (57). The abundance of chemokines involved in atherosclerosis corresponds to the functional complexity of pathogenesis.

## Chemokines and their receptors in coronary artery disease

Coronary artery disease (CAD), a key outcome of atherosclerosis, causes significant morbidity and mortality in populations around the world. While much is yet to be discovered, CCL2, CCL5, CXCL1, and CXCL12 are found to be involved in CAD (58–62). A study by Luciano-Mateo et al. has demonstrated that autopsy reports of recently deceased individuals from CAD had a higher level of CCL2 in their serum (63). Although they caution that their finding may not be representative and that it is just an observational study, there has been another study done by Li et al. that continues to build upon that report. They have shown that circulating CCL2 serum levels correlated with an increase in the risk of CAD (58, 59). CCL5-polymorphism has been linked to an increased risk of CAD (59, 64). While this shows a pathogenic role of CCL5 in CAD, there are contradicting reports from Gencer et al. that state CCL5 is responsible for halting immune cell function and infiltration, leading to reduced plaque formation and induction of CAD (61). CXCL1 is shown to be involved in recruiting macrophages and lesion progression (38). A study has shown that the gene expression of CXCL1 was increased in PBMCs from CAD patients when compared to the healthy controls (65). CXCL12 attracts immune cells and stem cells to wounded vascular endothelial cells (59, 61, 66). High levels of this chemokine are associated with CAD (66). While more research is necessary to better understand the roles that chemokines play during CAD, it is important to note that chemokine attraction of immune cells to the site of the vascular endothelium play a major role in plaque formation and thereby CAD.

## Chemokines and their receptors in heart failure

Heart failure is characterized by dyspnea, poor exercise tolerance, fatigue, and fluid retention caused by cardiac dysfunction. Heart failure (HF) affects 6.2 million American adults and projections estimate about 8 million adults will be affected by HF by 2030 (15, 67). Studies such as the RELAX, TIME-CHF, and ASCEND-HF trials have consistently shown the high prevalence of inflammation in heart failure (68–70). Chemokines, being a major player in the immune system, have been demonstrated to be consistently elevated in heart failure (71, 72) (**Figure 1**). Heart failure is classified based on reduced (HFrEF) or preserved (HFpEF) ejection fraction. In HFrEF, chemokine activation is driven primarily by myocardial injury, necrosis, and adverse remodeling especially early post-MI or primary cardiomyopathies, producing a surge in CCL2–CCR2, CCL5-CCR5, CX3CL1–CX3CR1, and CXCL8–CXCR2 signaling that recruits inflammatory monocytes and neutrophils into the injured ventricle (73–75). In contrast, HFpEF is characterized by systemic metabolic inflammation and microvascular dysfunction, where chemokines such as CCL2, CCL5, CXCL5, and CX3CL1 originate largely from adipose tissue, vascular endothelium, and perivascular macrophages rather than necrotic myocardium. While both phenotypes share chronic activation of monocyte/macrophage−related axes (e.g., CCL2–CCR2, CCL5-CCR5, and CX3CL1–CX3CR1), HFrEF exhibits acute, injury−centric chemokine induction, whereas HFpEF displays persistent, low−grade, metabolically primed chemokine signaling (76–78). Studies have shown that CCL2 has been expressed in both HFrEF and HFpEF and the proinflammatory role of CCL2 leading to adverse remodeling and cardiac dysfunction (79–81). In heart failure patients with reduced EF, increased CCL2 levels correlated with worsening of symptoms and poor systolic dysfunction (73). This finding was backed by Aukrust et al. whose study showed that CCL2 levels were inversely correlated with left ventricular EF (82). Moreover, higher CCL2 levels were linked to higher mortality rates in patients with advanced heart failure (83). CCL5 plays a notable role in the inflammatory processes in both HFrEF and HFpE. Inhibition of CCL5 led to improved cardiac function and survival in post-MI cardiac failure murine models (74). Similarly, in the ischemia/reperfusion murine models, intraperitoneal injections with CCL5 antagonists resulted in less oxidative stress and decreased cardiomyocyte death (84). CXCL1, CXCL5, and CXCL8 were increased in patients with congestive heart failure in a study conducted by Damas et al. (79). Recent study has shown that heart failure-specific fibroblasts contribute to the heart failure through a CXCL1-CRCR2 axis (85). With chemokines playing such a central role in the development and progression of heart failure, targeting these chemokines could impact the lives of millions worldwide.

## Chemokines and their receptors in arrhythmias

The heart contracts in a synchronized manner which is coordinated by a complex electrical system within the heart tissue. Normal sinus rhythm originates from the SA node and propagates via the His-Purkinje system to depolarize the ventricles in a systematic way to cause controlled muscle contractions. A disruption or irregularity in this electromechanical system leads to an arrhythmia. The heart is highly innervated by the autonomic nervous system (ANS) and the connection between the ANS and arrhythmias is well established. Chemokine signaling alters autonomic tone by driving inflammation, sympathetic hyperinnervation, and vagal suppression. This imbalance destabilizes cardiac electrophysiology, making arrhythmias more likely and more severe (**Figure 1**). A recent study has shown that increased resistin-like molecule gamma (RELMγ) from neutrophils attacks cardiomyocytes which causes ventricular tachycardia (86). Furthermore, a meta-analysis conducted by Wu et al. evidenced that patients with increased levels of inflammatory mediators were at higher risk of developing atrial fibrillation (AF) than the general population (87). CCL3 has been shown to stimulates the nervous system during antigenic challenge (88). Elmas et al. showed that the levels of CXCL8 and TIMP1 were significantly higher in MI patients with ventricular fibrillation (VF) as compared to those without VF (89). Also, CXCL8, CCL2, CCL4, and other pro-inflammatory mediators were found to be elevated in patients with arrhythmogenic right ventricular dysplasia (ARVD) as compared to controls (90). It is interesting to note that CXCL8 levels were higher in patients with permanent AF than in those with paroxysmal AF (91). CXCL9 was also found to have a direct impact on cardiomyocyte action potential duration and electrical stability of the heart (92). CXCR2 was shown to be involved in atrial fibrillation by driving monocyte infiltration (93). Thus, targeting these chemokines and their receptors could help to reduce the stimulation of the ANS which in turn reduce arrhythmia.

## Chemokines and their receptors in myocarditis

Myocarditis is characterized by inflammation of the heart muscle and is said to be caused by various infectious and non-infectious etiologies (94) (**Figure 1**). Among the infectious etiologies, viruses are presumed to be the most common pathogen. Viral myocarditis is acute onset and may resolve in response to viral clearance or progress to dilated cardiomyopathy. Some of the common causes of viral myocarditis include influenza A and B viruses, enteroviruses (coxsackie A and B), viruses of the Herpesviridae family, parvovirus B-19, adenoviruses, MERS-CoV, SARS-CoV, SARS-CoV-2, HIV, and hepatitis C virus (95). Viruses directly infect cardiomyocytes and activate TLR/RIG-I signaling pathways thereby increasing inflammatory mediators. Chemokines recruit neutrophils, monocytes and NK cells to clear the virus (95–97). CCL2 was also shown to be significantly elevated in mice with Coxsackievirus B3 (CVB3) induced myocarditis (98). From our studies, we have shown that the influenza infection increases CCL2, CCL3, CXCL9 and CXCL10 in the heart (99). Autoimmune disorders are also able to cause myocarditis due to chronic and progressive inflammation. Some of the autoimmune disorders that can cause myocarditis are systemic lupus erythematosus, systemic sclerosis, sarcoidosis, eosinophilic granulomatosis with polyangiitis, granulomatosis with polyangiitis, inflammatory myopathies, rheumatoid arthritis, myasthenia gravis, and autoinflammatory diseases (100). Autoantibodies against cardiac proteins, autoreactive T cells, and chemokine-mediated chronic inflammation are the primary mechanisms involved in auto-immune myocarditis (101). Studies in murine models of experimental autoimmune myocarditis (EAM) showed upregulation of CCL2 (102, 103). Blocking CCL2 with monoclonal antibodies resulted in reduced severity of autoimmune myocarditis (102). A study by Leuschner et al. revealed that siRNA silencing of CCR2 (siCCR2) led to reduced leukocyte progenitor trafficking in autoimmune myocarditis (104). These studies suggest that targeting these chemokines and their respective receptors may be beneficial for myocarditis. However, recently detrimental effects of CCL17 deletion have been shown in viral myocarditis via suppression of regulatory T cells (105).

Overall, a small set of shared chemokine axes, particularly CCL2–CCR2, CCL5–CCR5, and CX3CL1–CX3CR1 form a common inflammatory and vascular framework across atherosclerosis, myocarditis, heart failure, and arrhythmia. These pathways are involved in monocyte recruitment, endothelial activation, and fibroblast remodeling, but each disease engages them in distinct tissue contexts. Atherosclerosis uses these axes to drive leukocyte accumulation and smooth muscle activation; myocarditis amplifies monocyte and T cell infiltration; heart failure repurposes them for post−injury inflammation or metabolic–microvascular dysfunction; and arrhythmias emerge when chronic chemokine signaling remodels atrial or ventricular substrate. However, some chemokines play specific role based on the disease. In atherosclerosis, CCL19/21- CCR7 axis play a role in plaque regression (56). Similarly, in myocarditis, the presence of CCL17 is shown to be beneficial (105). Collectively, these studies suggest that the chemokines play both shared versus disease specific roles in cardiovascular diseases.

## Targeting chemokines and their receptors in cardiovascular diseases

Studies have shown that CCL2 plays an extensive role in cardiac remodeling and dysfunction. Targeted antagonism of the CCL2:CCR2 axis may prove to be promising in heart failure therapy. MLN1202, which is an anti-CCR2 monoclonal antibody, significantly reduced C-reactive protein (CRP) levels. As we know, CRP is a biomarker used to classify patients at high risk for atherosclerosis. However, the direct association between MLN1202 and adverse cardiovascular events are not yet studied (106). A meta-analysis of pre-clinical studies shown that targeting CCL2: CCR2 axis resulted in reduction in atherosclerosis lesion formation (107). Bindarit, which inhibits the expression of CCL2, CCL7, CCL8 and IL-12, significantly reduced in-stent late loss in CAD patients who underwent a PCI, but had no effect on the major adverse cardiovascular outcomes (108).

The interactions of CCL5 with CXCL4, CCL17, and HNP1 present interesting therapeutic targets. Rationally designed synthetic peptides, termed MKEY, CAN, and SKY were implemented to block the interactions of CCL5 with CXCL4, CCL17, and HNP1, respectively. This resulted in a reduction of atherosclerotic plaque size and a reduced ischemia-reperfusion injury in mice treated with the respective peptides. These examples illustrate that interactions of CCL5 with other proteins might be an interesting approach for the treatment of CVD, with less risk for immunologic side effects as a total functional blockade of CCL5 (109). Maraviroc, a CCR5 antagonist, is a common medication used in HIV patients. Patients with HIV are at an increased risk of developing atherosclerosis and unsurprisingly, it has been considered for the prevention of atherosclerosis. In mice, Maraviroc successfully reduced atherosclerotic plaque development (110). It is currently undergoing a Phase IV trial to study the effect of CCR5 antagonism on atherosclerosis in HIV patients. POL6326, a CXCR4 antagonist, is currently in a phase II trial on the effect on heart function and infarct size in patients who underwent reperfusion after an ST Elevation Myocardial Infarction (STEMI). In the phase II STOP-HF trial, a single endocardial dosing of CXCL12 coding plasmid (JVS-100) improved cardiac function at 12 months after treatment in patients with left ventricular ejection fraction (EF) <26% post MI (111).

Despite strong evidence showing promising results targeting chemokines in cardiovascular diseases, a major barrier is the redundancy within the chemokine system. Multiple ligands signal through the same receptor, and many receptors respond to several ligands, allowing inflammatory recruitment to persist even when a single axis is blocked. In parallel, compensatory signaling rapidly restores leukocyte trafficking through alternative pathways including upregulation of CX3CL1–CX3CR1 or CCL5–CCR5 axis when CCR2 is inhibited—preserving inflammatory response within the myocardium or vasculature. A further limitation of current chemokine-based therapies is that the field has only recently begun to explore cell-targeted delivery strategies, such as nanoparticle-mediated monocyte targeting and ligand-directed approaches. Addressing these barriers will be essential for designing next-generation chemokine-directed therapies in cardiovascular diseases.

## Conclusion

Chemokines play a role in cardiovascular diseases by recruiting immune cells to the sub-endothelium influencing plaque stability, plaque progression, adverse remodeling, and cardiac dysfunction in heart failure, arrhythmias, and myocarditis. The pathophysiologic heterogeneity of these diseases is a major barrier that hinders the development of new therapeutics. For example, patients with diabetes mellitus or chronic kidney disease have an increased risk of CVD, partly caused by increased and altered inflammatory processes in these patients. Unravelling comorbidity-associated alterations is essential for successful clinical translation in defined patient groups, supporting more personalized treatment approaches. Similarly, infection induced inflammation increases the outcome of the disease severity. Inhibiting the immunological function of these proteins may also be detrimental. Most studies have focused on suppressing inflammation rather than promoting its active resolution, a biologically distinct process mediated by specialized pro-resolving mediators, macrophage reprogramming, and coordinated clearance of inflammatory cells. Failure to distinguish suppression of inflammation from resolution can impact necessary repair responses or incompletely extinguish chronic inflammation. Therefore, specific treatment approaches are required to tackle these issues in developing chemokine-based therapeutics in cardiovascular diseases.
